# Spectroelectrochemical Studies of Oxygen Evolution Reaction Kinetics for Surface-Incorporated Iron in Nickel Oxyhydroxide Electrocatalysts

**DOI:** 10.1021/acscatal.5c09080

**Published:** 2026-03-19

**Authors:** Yifeng Wang, Liam P. Twight, Nicole A. Sagui, Minkyoung Kwak, Shannon W. Boettcher, Benjamin S. Moss, Ifan E. L. Stephens, James R. Durrant, Reshma R. Rao

**Affiliations:** † Department of Materials, 4615 Imperial College London, London SW7 2AZ, U.K.; ‡ Department of Chemistry and Biochemistry, 1438 University of Oregon, Eugene, Oregon 97403, United States; § Department of Chemical & Biomolecular Engineering and Department of Chemistry, University of California, Berkeley, California 94720, United States; ∥ Energy Storage and Distributed Resources Division, Lawrence Berkeley National Laboratory, Berkeley, California 94720, United States; ⊥ Department of Chemistry, Imperial College London, London W12 0BZ, U.K.; # Centre for Processable Electronics, Imperial College London, London W12 0BZ, U.K.; ∇ Grantham Institute − Climate Change and the Environment, Imperial College London, South Kensington Campus, London SW7 2AZ, U.K.

**Keywords:** water oxidation, catalysis, spectroelectrochemistry, kinetics, mechanism, nickel oxide

## Abstract

Ni_*x*_Fe_1–*x*_O_*y*_H_*z*_ is the state-of-the-art catalyst for the oxygen evolution reaction (OER) in alkaline water electrolyzers; however, understanding the impact of Fe on the active sites, reaction mechanism, and consequently intrinsic activity has been under intense debate. In this work, *operando* UV–vis spectroscopy was used to investigate Fe-free NiO_*x*_H_*y*_ and NiO_*x*_H_*y*_ with Fe selectively incorporated onto the surface. At oxygen-evolution potentials, similar oxidized nickel states were present before and after the Fe incorporation, with negligible changes in their redox potentials. However, the discharge kinetics of the Ni states show a substantial acceleration after the introduction of Fe, consistent with an increase in OER kinetics upon Fe incorporation and formation of active Ni–Fe species. Using optical spectroscopy, we determined the intrinsic reaction time constant per surface Fe site is <0.1 s, which is 2 orders of magnitude faster than Ni sites not in proximity to surface Fe sites (∼10 s), and also an order of magnitude faster than Ni sites in pure NiO_*x*_H_*y*_ (∼1 s). Consequently, we propose that the OER occurs via charge accumulation primarily on Ni centers in these catalysts, followed by hole transport to the surface Fe species where oxygen evolution occurs.

## Graphical abstract

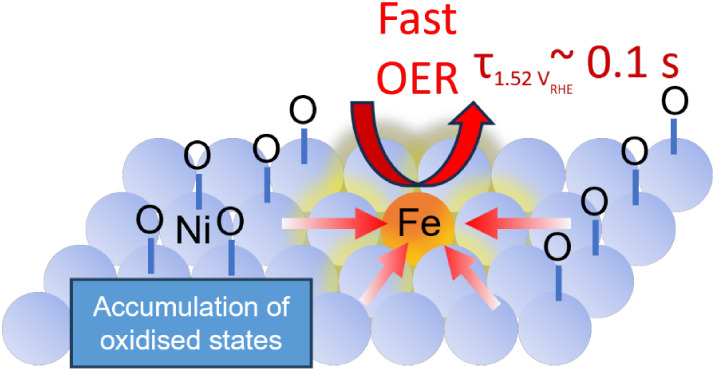

## Introduction

The oxygen evolution reaction (OER) has slow kinetics and limits the efficiency of water splitting to make hydrogen fuel. Fundamental understanding of the reaction mechanisms governing OER can accelerate the design of improved catalysts.^1–3^ Nickel-based catalysts, and particularly Ni_*x*_Fe_1–*x*_O_*y*_H_*z*_, have the highest recorded performance for OER under alkaline conditions^4–8^ and hence have been widely employed in commercial electrolyzers.^2,9^ Small amounts of Fe, even a few ppm in the electrolyte, can be incorporated into the catalyst and have a dramatic effect on the OER activity.^6,10^

The exact role that incorporated Fe plays in the NiO_*x*_H_*y*_ framework to increase the OER activity remains under study. Mechanistically, it has been suggested that (1) Fe causes structural distortion and charge effects on the host NiO_*x*_H_*y*_ using through-film in situ conductivity, as suggested by Trotochaud et al.,^6^ and by Etxebarria et al.^11^ with X-ray absorption spectroscopy (XAS) as well as *operando* Raman spectroscopy; (2) Fe acts as the dominant active site, with evidence provided by Friebel et al.^12^ and Song et al.^7^ using *operando* XAS and density functional theory (DFT) calculations; (3) both Ni and Fe act as the active site via a cooperative effect, as suggested by Francás et al.^13^ using *operando* UV–vis spectroscopy. Although consensus remains obscure regarding the overall impact of incorporated Fe and whether Ni or Fe sites function as the primary catalytic sites, it has been widely reported that the optimal amount of Fe leading to the highest overall OER activity is ∼25%.^6,14^ Regardless, it seems that both Ni and Fe are potentially involved in the reaction and probably together contribute to the formation of distributions of active sites within the heterogeneous catalyst.

Apart from the fraction of Fe, the methods used to incorporate Fe affect the OER activity. Both the unintentional introduction of Fe (from impurities in the electrolyte) and the intentional introduction of Fe by co-deposition improve the activity of NiO_*x*_H_*y*_.^6^ Fe can be introduced during sample preparation via a range of synthesis methods, including electrodeposition,^6^ e-beam evaporation,^11^ magnetron sputtering,^15^ size-selected deposition of nanoparticles,^16^ and solution combustion.^17^ Our recent work^18^ has shown a method of surface-restricted Fe incorporation using a chronoamperometry technique (Fe/Ni atomic ratio from 0 to 20%) with controlled amounts of Fe in the electrolyte (down to 0.1 ppm). The resulting amount of incorporated Fe was determined by inductively coupled plasma mass spectrometry (ICP-MS), normalized by the electrochemical surface area of the host NiO_*x*_H_*y*_. We have reported that with an increasing amount of Fe incorporated specifically at the surface, from 0.4 to 2.9 ng cm^–2^_ECSA_, the turnover frequency per iron center (TOF_Fe_, η = 300 mV, per O_2_ evolved) rises significantly from ∼2 to ∼10 s^–1^.^18^ Considering the low loading of the incorporated Fe and the dramatic improvement in the turnover frequency, it is reasonable to believe that surface-active Fe-containing species take part in the reaction.

Probing the intrinsic catalytic activity of (electro)­catalysts and the mechanism for the OER is challenging since the potential-dependent density of redox-active states is difficult to determine. Various approaches have been used in the literature to estimate the electrochemical surface area, including redox wave integration and double-layer capacitance to determine the electrochemically active surface area, Brunauer–Emmett–Teller (BET) analysis, and microscopy.^19^ Some reports assume that all of the Ni and Fe in the catalyst are active toward OER, while others use the number of nickel sites involved in the redox reaction at ∼1.35 V_RHE_ to estimate the density of active sites.^20,21^ Our recent results on Ni_*x*_Fe_1–*x*_O_*y*_H_*z*_,^13^ NiO_*x*_ films,^16^ and NiO nanoparticles^17,22^ have demonstrated an absence of correlation between the charge passed during the redox transition prior to OER and the oxidized species present at oxidizing potentials. On the other hand, experiments on mass-selected Ni_*x*_Fe_1–*x*_O_*y*_H_*z*_ nanoparticles showed that only the surface layer participated in the reaction, while ∼3 atomic layers of the near-surface region of the nanoparticles are redox active.^15^ The challenges of deconvoluting the currents from multiple pseudocapacitive redox transitions and the OER current are further exacerbated for highly heterogeneous surfaces, where minority sites can be solely responsible for the catalytic activity. For example, Mefford et al. have shown that the edge sites on Co­(OH)_2_ dominate the OER activity;^23^ likewise, Kibsgaard et al. showed that the HER activity is dictated by the edge sites of MoS_2_.^24,25^ Eom et al. have also demonstrated with doped MnO_*x*_ that placing the active layer over different subsurfaces can alter the activity toward oxygen reduction.^26^ Given these ambiguities in determining the active centers at oxidizing potentials, quantitative determination of the reaction kinetics in the presence of surface-incorporated Fe and the mechanism governing the improvement in the OER activity remains elusive.

We have recently shown that *operando* UV–vis spectroelectrochemistry is able to quantitatively deconvolute redox species populations as a function of potential in-situ, which can be used to estimate the intrinsic TOF of these species driving OER.^17,27,28^ In this study, we extend the same *operando* spectroelectrochemical (SEC) methods to investigate three different NiO_*x*_H_*y*_ catalysts: NiO_*x*_H_*y*_, NiO_*x*_H_*y*_ with Fe incorporated and restricted to the surface of the NiO_*x*_H_*y*_ film, and Ni_0.5_Fe_0.5_O_*x*_H_*y*_ with Fe incorporated into the bulk structure through co-deposition.

Compared with previous *operando* optical or X-ray absorption spectroscopy (XAS) studies,^13,16,29,30^ where the means of Fe incorporation was not well-defined, resulting in Fe being located both in the bulk and absorbed onto the surface of NiO_*x*_H_*y*_, and hence making the role of Fe in both cases indistinguishable, our approach provides a systematic and internally consistent framework to assess how surface Fe influences Ni redox kinetics. Previous reports also do not quantify the TOF relative to dynamically probed active site populations,^16,29,30^ which is enabled by our optical spectroscopy measurements herein. Our findings reveal that the surface incorporation of Fe into NiO_*x*_H_*y*_ does not lead to significant changes in the UV–vis absorption difference spectra under potentiostatic control. On the other hand, for the sample with co-deposited Fe, the spectra show distinctive features related to Fe-centered redox.^13^ This finding suggests that the species accumulated under anodic bias are oxidized Ni species before and after the surface-restricted Fe, without modification of the bulk Ni. However, a remarkable increase in the rate of open-circuit discharge of these accumulated Ni species (from NiOO to NiOOH) at >1.4 V_RHE_ was observed upon surface incorporation of Fe. This suggests that the significant increase in oxygen evolution kinetics is driven by the introduction of surface Fe-containing species. By determining the density of oxidized Ni centers and the intrinsic rate of reaction, we propose that the enhanced OER activity arises from Fe-facilitated charge transfer, where accumulated NiOO species act as charge reservoir sites that deliver holes to a small number of catalytically active Fe surface centers. This is the first time our *operando* studies have shown that water oxidation kinetics can be dominated via minority sites present on the surface, where the majority of the active sites act simply as charge reservoirs for the surface minority sites.

## Results and Discussion

Electrodeposited Fe-free Ni­(OH)_2_ on fluorine-doped tin oxide (FTO) glass and Fe-free 1.0 M KOH were prepared using the protocol developed by Trotochaud et al.^6^ The films show a sheet-like morphology (Figure S1), and the Fe 2p XPS (Figure S2) spectra, as well as the near-edge X-ray absorption fine structure in total electron yield mode (Figure S3), indicate the absence of Fe in the samples, which is consistent with earlier studies.^6,13^ Film thickness was measured by profilometer, which was ∼24.2 nm for the NiO_*x*_H_*y*_ and ∼24.1 nm for co-deposited Ni_0.5_Fe_0.5_O_*x*_H_*y*_ (Figure S4). The Fe-spiked samples were prepared using the protocol by Ou et al.^18^ by holding the Fe-free NiO_*x*_H_*y*_ sample under chronoamperometry at 1.55 V_RHE_ for 30 min (Figure S5) in ∼10 mL of 1.0 M Fe-free KOH containing ∼0.1 ppm Fe^3+^.

Transition metals present in the electrolyte can be incorporated into the bulk NiO_*x*_H_*y*_ scaffold under extensive cycling or deliberate co-deposition. Transition metals incorporated within the bulk NiO_*x*_H_*y*_ have been found to modify its electronic structure, which results in the shift of the Ni­(OH)_2_ + OH^–^ → NiOOH + H_2_O + e^–^ (T2) redox peak compared with pure NiO_*x*_H_*y*_.^6,17,18^ Furthermore, the shift in the peak position increases with increasing cycle number, as cycling induces a dissolution–redeposition process and the amount of incorporated ions increases.^31^ However, as demonstrated in our previous work, holding pure NiO_*x*_H_*y*_ at constant potential in a transition metal-containing electrolyte has been demonstrated to restrict the incorporation to the surface of NiO_*x*_H_*y*_ scaffold with the majority of the bulk Ni sites unaltered.^18^ In our study, Fe was incorporated at 1.55 V_RHE_ via chronoamperometry instead of cycling in Fe-containing electrolyte, and spectroelectrochemical measurements were immediately conducted by scanning from 1.13 to 1.55 V in a staircase chronoamperometry protocol. The positive shift in the Ni­(OH)_2_ + OH^–^ → NiOOH + H_2_O + e^–^ (T2) redox peak, as previously proposed when Fe is introduced by cycling in Fe-containing electrolyte or co-deposition,^6^ was not observed here, yet a remarkable increase in OER activity was recorded (Figure 1A). This is supported by the scanning electron microscope (SEM, Figure S1) image taken before and after the Fe-spiking process, as the introduction of Fe into NiO_*x*_H_*y*_ has been shown to modify its structure.^6,11^ As seen in Figure S1, no significant structural change is observed in the sample before and after the Fe-spiking process, and the majority of the NiO_*x*_H_*y*_ remains unchanged, indicating that the Fe introduced is restricted to the surface of NiO_*x*_H_*y*_. This was further confirmed by the near-edge X-ray absorption fine structure (NEXAFS, Figure S3) collected in total electron yield (TEY, surface-sensitive) and total fluorescence yield (TFY, bulk-sensitive). Previous extended X-ray absorption fine structure (EXAFS) on similar samples showed Fe-oxo clusters forming on the surface with coordination numbers ∼5, which are larger than a dimer adsorbed on the surface (CN 3) but smaller than an extended crystal (CN 6).^32^ Furthermore, the electrochemical signature does not exhibit features characteristic of bulk FeO_*x*_ phases.^13^ Taken together with the absence of bulk FeO_*x*_ electrochemical signals,^13^ it could be concluded that Fe was successfully and dominantly incorporated onto the surface NiO_*x*_H_*y*_ matrix rather than forming long-range segregated FeO_*x*_ islands.^18^

**Figure 1:**
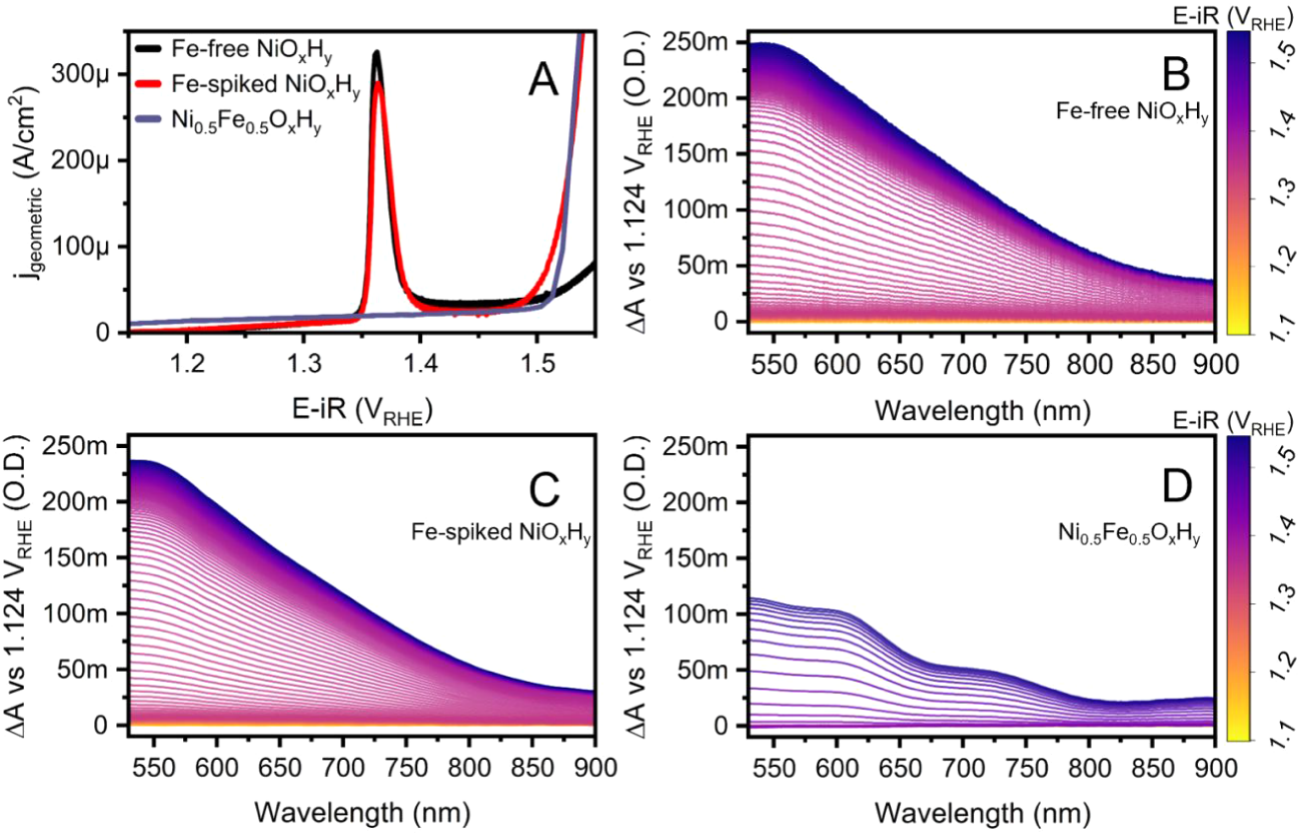
(A) *iR*-corrected linear sweep voltammogram (1.0 M Fe-free KOH, 1 mV/s from 1.13 to 1.55 V_RHE_, Hg/HgO reference electrode filled with 1.0 M Fe-free KOH, Pt wire counter electrode, room temperature): Fe-free NiO*_x_* H*_y_* (black), Fe-spiked NiO*_x_* H*_y_* (red), and Co-deposited Ni_0.5_Fe_0.5_O*_x_* H*_y_* (purple, done at 10 mV/s). *Operando* UV–vis spectra from ∼1.1 V_RHE_ to ∼1.55 V_RHE_ for (B) Fe-free NiO*_x_* H*_y_*, (C) Fe-spiked NiO*_x_* H*_y_*, and (D) Co-deposited Ni_0.5_Fe_0.5_O*_x_* H (done at 10 mV/s). i: current; *j* is normalized with the geometric area of the electrode; *R*: uncompensated resistance.

*Operando* UV–vis spectroscopy can be used to track the oxidized species formed at oxidative potentials.^13,17,28,30,33^ Figure 1B–D shows *operando* UV–vis difference spectra as a function of potential for Fe-free NiO_*x*_H_*y*_, Fe-spiked NiO_*x*_H_*y*_, and co-deposited 50% Fe NiFeO_*x*_H_*y*_. There are no significant differences between the spectral shape in the Fe-free and Fe-spiked samples, although there is a small absorption amplitude loss after Fe-spiking, which might be caused by minor delamination of the film when deposited onto an FTO substrate. This was verified by the integration of the T2 oxidation wave and spectroscopic measurement of the population of Ni species (Figure S6). On the other hand, the 50% Fe sample has very different optical absorption features, consistent with our previous study.^13^

These difference spectra can be deconvoluted into three component spectra with unique spectral shapes, as shown in eq 1 (see Supporting Information for detailed analysis):

1 $$\Delta A\left(\lambda ,U\right)=\underset{redox\;transitionsT_{i}}{\sum }Q_{i}\left(U\right)\times \Delta \alpha_{i}\left(\lambda_{peak}\right)\times \overset{¯}{\Delta \alpha_{i}\left(\lambda \right)}$$

where Δ*A*(λ,*U*) represents the measured change in absorbance, subtracting the signal from the resting potential of the sample (1.124 V_RHE_); $\overset{¯}{\Delta \alpha_{i}\left(\lambda \right)}$ is the differential coulometric attenuation coefficient spectra normalized at its wavelength of maximum absorbance, which can be interpreted as “component spectrum” of a distinct redox transition *T_i_*; and Δ*α_i_* (λ*_peak_*) refers to the change in optical signal per C/cm² of charge passed onto the sample, evaluated at the wavelength of maximum absorbance for the corresponding redox transition.

Additionally, changes in the population of species $\frac{\partial Q_{i}}{\partial U}$ can be obtained using the fitting, which is in the form of ∂Q∂U . The scan rate of the measurement, which is 1 mV/s, has the form of ∂U∂t . Hence, the charging current of a redox transition can be approximated as

$$j_{charging}=\frac{\partial Q_{i}}{\partial U}*scan\,\;rate$$ 2

Here, we identified three $\overset{¯}{\Delta \alpha_{i}\left(\lambda \right)}$ which are assigned to redox transitions 1, 2, and 3 in ascending order of the potential window where these spectra appear (Figure 2A,B), following our previous work.^13^ At early potential ranges (<1.33 V_RHE_), T1 is convoluted with the capacitive charging^34^ and is not shown due to its small magnitude (Figures S7-S9). Based on previous studies, all three redox transitions have been attributed to one-electron reactions. Specifically, redox transition 1 (T1) is assigned to the oxidation of defect species to Ni­(OH)_2_, redox transition 2 (T2) to Ni­(OH)_2_ + OH^–^ → NiOOH + H_2_O + e^–^, and redox transition 3 (T3) to NiOOH + OH^–^ → NiOO + H_2_O + e^–^,^5,12,29,35,36^ where the NiOO species can have a slight negative charge.^35^ The species formed after T2 are henceforth represented as NiOOH, and the species formed after T3 are represented as NiOO. The potential windows of these changes are consistent with previous work, where the oxidation state of Ni is determined by XAS and Raman, further supporting our assignment.^5,12,35^

**Figure 2:**
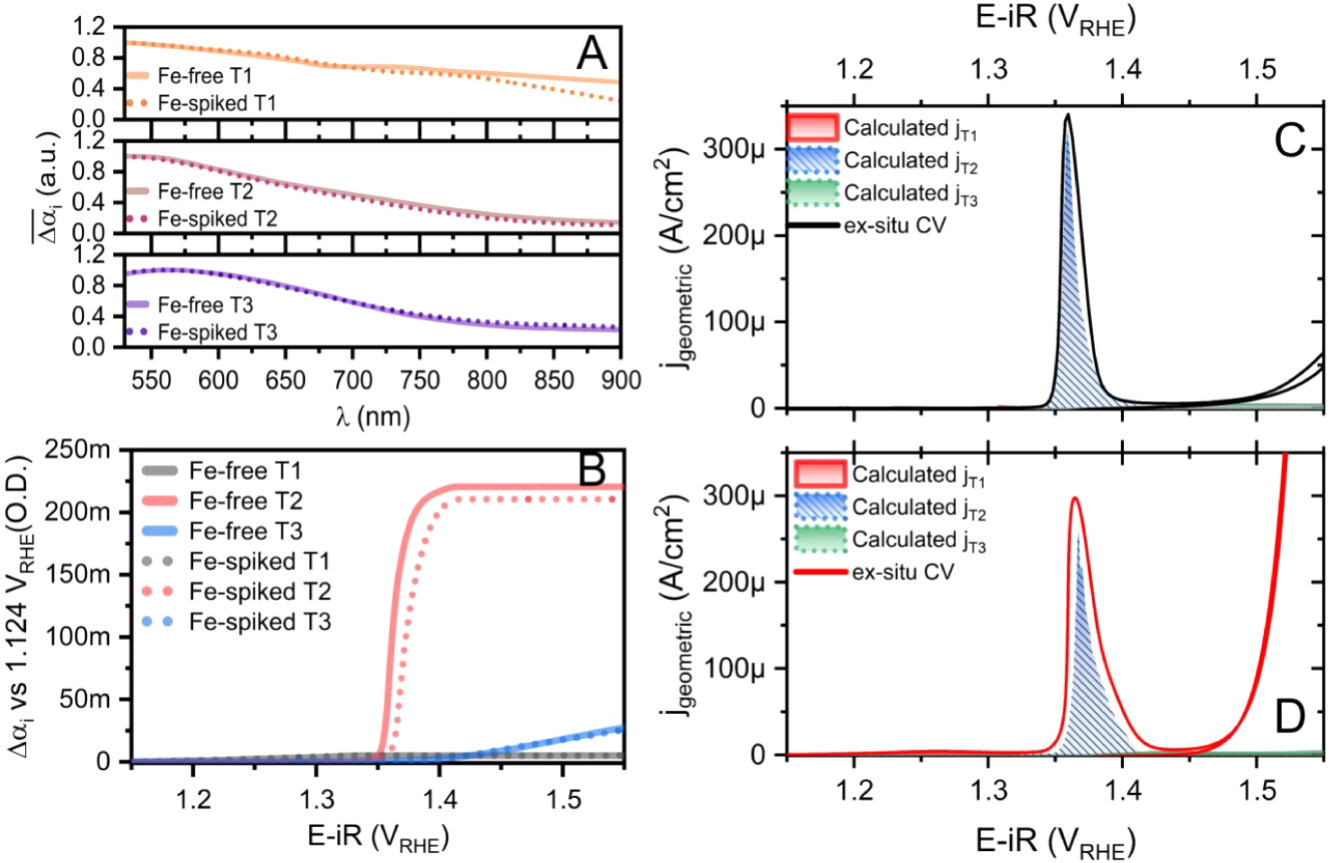
(A) The normalized differential coulometric attenuation coefficient spectra $\overset{¯}{\Delta \alpha_{i}\left(\lambda \right)}$ of Fe-free and Fe-spiked NiO*_x_* H*_y_* for redox transitions one, two, and three (T1–3). (B) The deconvoluted change in absorbance versus potential for each redox transition Δ*α_i_* (λ*_peak_*). The calculated charging current densities plotted against the cyclic voltammogram at 10 mV/s for (C) Fe-free and (D) Fe-spiked NiO*_x_* H*_y_*.

As seen in Figure 2A, there is no significant difference between the component spectra before and after the Fe spiking, suggesting that the chemical nature of the accumulated oxidized states is similar. By contrast, for the co-deposited Ni_0.5_Fe_0.5_O_*x*_H_*y*_ (Figure 1D), in addition to the absorption peak at ∼600 nm, another absorption peak at ∼700 nm was observed, consistent with Francás et al.,^13^ who showed that changing the fraction of Fe in the sample from 5% to 50% alters the absorption spectra, suggesting a change in the active center from Ni to Fe. Figure 2B–D and Figures S6–S15, on the other hand, show that Fe spiking does not result in a significant difference in the magnitude of redox transitions (as seen from the similar magnitude of Δ*α_i_* against potential for both the Fe-free and the Fe-spiked samples in Figure 2B), which could also be verified by the redox peak integration, which should be independent of the spectroscopy (Figure S6 and Figure S13B). Furthermore, the onset potentials of redox peaks for the formation of NiOOH and NiOO, as seen in Figure 2B are similar before and after Fe spiking, suggesting that the energetics of oxo binding on nickel sites are similar before and after Fe spiking in this case. This is contrary to our previous results on doped NiO,^17^ IrO*_x,_* ^27^ and CoO_*x*_H*_y_,* ^28^ where changes in active site binding energetics were determined from the deconvoluted optical spectra. Therefore, it can be concluded that in this particular way of introducing surface-restricted Fe, Fe spiking does not induce a significant detectable change in the nature of the observable species accumulated as a function of potential, as well as the energetics of these oxidized Ni centers. It is possible that the Ni sites in proximity to the surface Fe could have changes in their electronic structures, but due to its small fraction and small amount of Fe, more sensitive techniques are required to track the changes of these extremely minority Ni sites. Yet, the dominant Ni sites and their redox appear largely unchanged upon Fe incorporation.

To determine the OER reaction kinetics before and after Fe spiking, we performed an open-circuit decay experiment. Our previous spectroelectrochemical studies,^13,17,27,28^ inspired by Conway et al.,^37^ demonstrated that the initial rate of decay reflects the intrinsic kinetics of the individual redox states. Initially, the sample was maintained at a lower potential for 30 s before being switched to and held at a more oxidizing potential for 30 s, allowing for the accumulation of oxidized nickel states, and then the open-circuit potential was monitored for another 30 s. After opening the circuit, we tracked the time-resolved changes in the optical signal at 560 nm, where the peak absorption wavelength of NiOO species is located (Figure 2A). The time-resolved absorption signal was then normalized by its maximum value to yield a normalized plot against time (Figure 3A).

**Figure 3:**
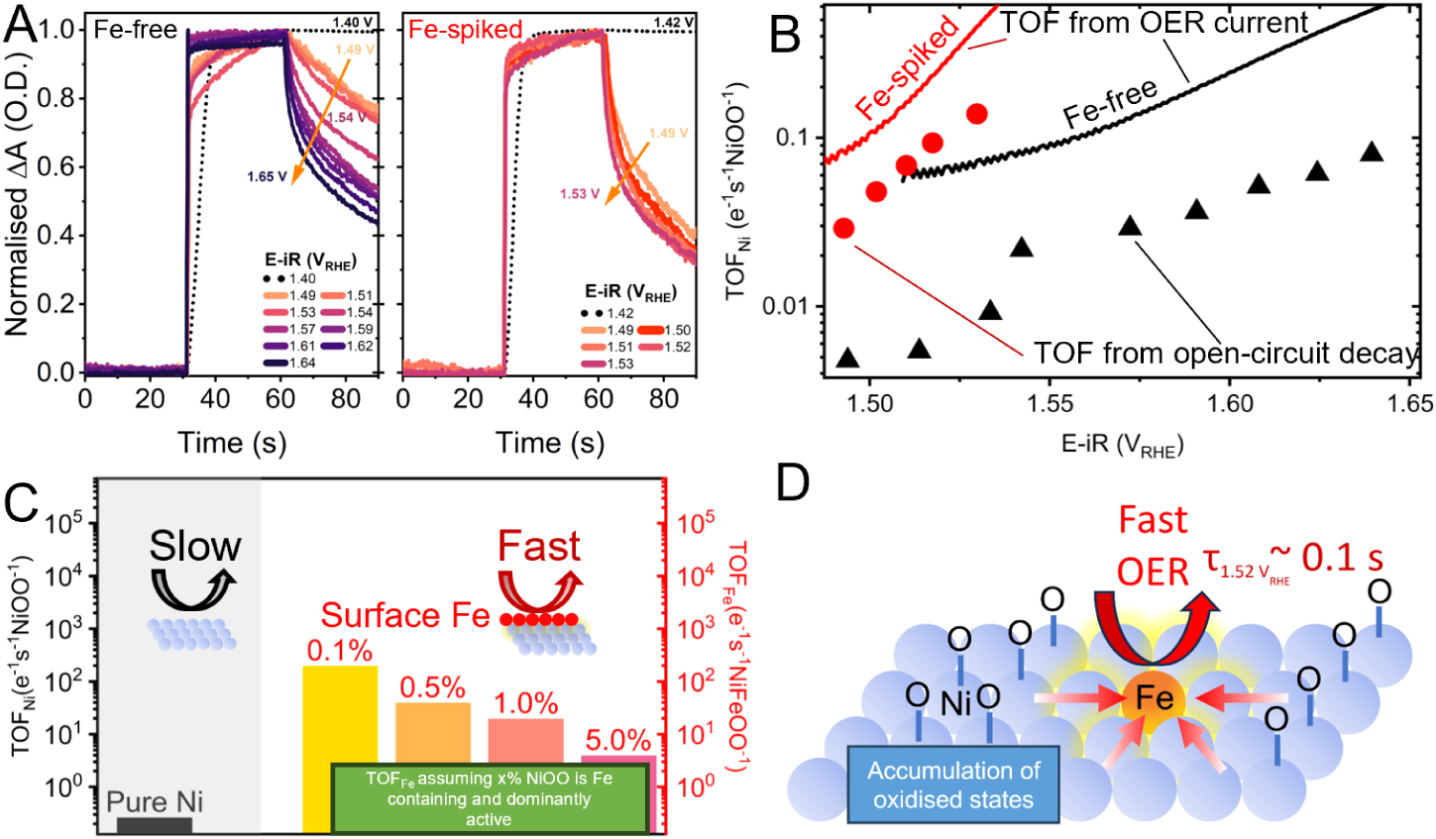
Normalized time-resolved potential decay for (A) Fe-free NiO*_x_* H*_y_* and Fe-spiked NiO*_x_* H*_y_*, intensity measured at 560 nm. The potential applied between 0 and 30 s was 1.42 V_RHE_ and the potentials applied between 30 and 60 s, which are increasingly higher across each experiment, are denoted in the figure legend. However, for the first decay, the potential applied in the first 30 s is 1.12 V_RHE_ and the subsequent 30 s is 1.40 V_RHE_ (shown as a black dotted line). From 60 s onward, the circuit is opened, and the system is allowed to decay. (B) Turnover frequency (TOF) of Fe-free and Fe-spiked NiO*_x_* H*_y_* calculated from measured current and the potential decay, normalized with the amount of NiOO measured; see more details in eqs 3 and 4 and Supporting Information. (C) TOF_Ni_ of the Fe-free NiO*_x_* H*_y_* and TOF_Fe_ of the Fe-spiked NiO*_x_* H*_y_* at 1.52 V_RHE_, calculated by assuming that only 0.1%, 0.5%,1%, and 5% of the observed Ni states contain Fe and are dominantly active. (D) Schematic showing the proposed mechanism for OER on surface-incorporated Fe NiOOH. Oxidized states are accumulated on the Ni centers at OER-relevant potentials, although the OER reaction occurs on surface Fe-containing species, with TOF_Fe_ > 10 s^–1^ NiFeOO^–1^ at 1.52 V_RHE_.

As seen from Figure 3A, for the Fe-free NiO_*x*_H_*y*_, at 1.40 V_RHE_, where a small fraction of NiOO species exists and the surface is dominated by NiOOH species (determined from Figure 2B), the optical signal barely decays when the circuit is opened, indicating that the few NiOOH species present turn over very slowly. However, in the potential region with an increasing population of NiOO, the initial rates of decay in the signal accelerate with increasing potential, indicating that TOF is correlated with NiOO coverage (Figure S16-S17). However, the role of oxidized states in driving OER, either via a potential-independent activation energy^13,16,17,22,38–40^ or via interaction between adjacent sites, as we observed for IrO_*x*_ ^27,41^ and CoO_*x*_H_*y*_,^28^ is beyond the scope of the current work. It is also worth noting that, as the surface Fe does not produce any noticeable change in the absorption spectrum, we are not directly observing the energetics of Fe species, while we are observing those of the host NiO_*x*_H_*y*_. After Fe spiking, the initial decay rate accelerates at a significantly lower potential, which agrees with the electrochemical measurements showing an increase in OER activity. The faster decay kinetics being triggered at less positive potentials in the presence of surface-incorporated Fe suggests that these Fe-containing species (which may include surface FeO_*x*_ clusters, as proposed previously^18^ that are formed upon Fe incorporation) are responsible for the faster OER kinetics.

To quantitatively determine the change in kinetics, TOF (turnover frequency) per accumulated Ni center is calculated as follows:

$$TOF_{j,Ni}=\frac{j_{\mathrm{geometric}}-j_{T3}}{Q_{NiOO}\times e}$$ 3

where *j*_geometric_ is the geometric current density in A/cm^2^, *j*_T3_ is the charging current in A/cm^2^ for the formation of NiOO as shown in Figure 2C,D,*Q_NiOO_* is the amount of NiOO calculated from optical spectroscopy in sites/cm^2^, and *e* = 1.60 × 10^–19^ C which is the elementary charge.

The same calculation can be done in a similar way on the optical decay by using the initial rate of decay:

TOFPD,Ni=δΔAδtαNiOO×e 4

where δΔAδt is the initial rate of decay in the optical signal, α*_NiOO_* is the Coulombic differential attenuation coefficient calculated from the optical spectroscopy in C^–1^cm^2^ (see Supporting Information). Note that in both calculations, the faradic efficiency is assumed to be unity. About a 1 order-of-magnitude increase in TOF_Ni_ is achieved after the Fe spiking (Figure 3A,B) at matched potentials (and therefore matched surface densities of oxidized Ni species), confirming the acceleration of the overall kinetics of Ni discharge redox. It is important to note that both *j*_T3_ and *Q_NiOO_* are obtained from spectral fitting and therefore may carry some uncertainty. Nonetheless, our previous work and other literature have demonstrated that this fitting model is physically meaningful.^29,30,30,42^ By contrast, parameters, such as δΔAδt and α*_NiOO_*, are independent of spectral decomposition; they depend solely on the experimentally observed decay kinetics at specific wavelengths. The good agreement between *TOF_j,Ni_* and *TOF_PD,Ni_* across the examined potential range provides strong validation for the reliability of our analysis and supports the physical soundness of the proposed model.

Notably, in our previous work, where 10% Mn, Co, Fe, and Zn were mixed in NiO, a shift in the redox peak was observed, signifying the dominant role of the Fe sites in modifying the electronic structure of redox-active Ni.^17,22^ However, in this work, the redox potentials and spectral signatures of Ni remain essentially unchanged upon Fe incorporation (Figure 2), indicating that the binding energetics of oxygenated species are largely unchanged. Therefore, the acceleration in discharge kinetics at lower potentials of Ni sites can be attributed to the formation of minority Fe-containing clusters on the surface, suggesting that OER proceeds preferentially at Fe-containing surface motifs, while Ni acts primarily as a charge reservoir.

Assuming fast equilibrium of charges between Ni and the Fe sites and a dominant equilibrium constant toward Ni (eqs 5 and 6), higher OER rates on minority surface Fe-containing species consequently lead to faster discharge of oxidized states accumulating on the surface. Specifically, considering the overall small fraction of Fe sites compared to bulk Ni, the kinetics on the Fe sites must be significantly faster than the discharge rate of Ni sites. Ou et al’s^18^ DFT calculations suggest that highly active Fe sites are able to share and stabilize oxidative holes between other neighboring Fe sites and with the host NiO_*x*_H_*y*_; therefore Fe–O–Fe dimers could be highly active. The similarity of the redox potentials of the Ni sites before and after Fe incorporation, despite the significant increase in reaction kinetics, supports the notion that newly formed surface Fe-containing species are highly active and lead to the fast discharge of the Ni sites.

However, it appears that Fe sites do not show potential-dependent optical absorption, possibly due to their lower concentration, which is beyond the detection limit of the instrument. The minimal detection limit requires at least ∼3.2% redox-active Fe in the sample at 1.55 V_RHE_ (see Supporting Information), which is larger than what we expected for the surface-restricted Fe. The UV–Vis absorption features of possible Fe^3+^/Fe^4+^ species are weak and overlap strongly with the intense ligand-to-metal charge transfer bands associated with oxidized Ni species; hence, more sensitive techniques (*operando* X-ray absorption spectroscopy,^30^ Mössbauer spectroscopy^43^) may be required to track the evolution of the very small population of Fe species under OER in this case.

Following the model described above, we assume that surface Fe-containing species act as reaction centers, with the Fe sites and the surrounding oxidized Ni states being energetically equivalent, thereby facilitating charge transfer. Under these assumptions, the charge equilibrium between the Ni and Fe sites within the surface Fe-containing species can be described by the equations below:

5 $$Ni^{x+}+Fe^{z+}\overset{K}{\to }Ni^{(x-1)+}+Fe^{(z+1)+}$$

6 $$4Fe^{(z+1)+}+4OH^{-}\overset{rds,k_{OER}}{\to }4Fe^{z+}+O_{2}+2H_{2}O$$

where *k_OER_* is the intrinsic rate constant for water oxidation on Fe sites (for simplicity, this is assumed to be effectively first order), and *K* is the equilibrium between oxidized NiOO sites and Fe sites. Presumably, these redox transitions would be coupled to the adsorption of reaction intermediates. For simplicity, we assume the enthalpies of Ni and Fe sites are similar, such that *K* is given by the ratio between Ni and Fe sites on the surface, i.e., for 1% Fe sites on the surface, *K* ∼ 0.01. Assuming a fast equilibrium between Ni and Fe sites, with the OER being the rate-determining step, this simple kinetic model allows estimation of the intrinsic TOF for OER of oxidized Fe species (TOF_Fe_) from the experimentally observed TOF of the spectroscopically dominant oxidized Ni species (TOF_Ni_) from

7 $$TOF_{Fe}=\frac{1}{K}\times TOF_{Ni}$$

Where the equilibrium constant *K* is determined by the ratio of Ni–Fe sites to Ni sites on the electrode surface. We note that, in our proposed fast equilibrium model, this equilibrium constant is independent of applied potentials. As shown in Figure 3C and Figure S18, at 1.52 V_RHE_, the TOF per oxidized Ni state is ∼0.2 s^–1^. However, if the OER occurs dominantly on surface Fe-containing species, as proposed herein, the intrinsic TOF of these Ni–Fe sites can be estimated to be ∼20 s^–1^ (assuming 1.0% Fe sites on the surface), ∼40 s^–1^ assuming 0.5%, and ∼200 s^–1^ assuming 0.1% Fe sites on the surface. Although some oxidized Ni sites will still be OER active, their contribution would be small (assuming their TOF_Ni_ remains unchanged upon Fe incorporation), and Ni sites act primarily as a charge reservoir under the conditions studied, while Fe-containing sites dominate the observed kinetic response.

It is worth noting that, since some Fe may be present in the substrate (FTO glass), ICP-MS analysis has been challenging. Hence, the amount of Fe incorporated can only be estimated based on previous work by Ou et al.,^18^ where the same Fe incorporation condition was used. Using the assumed percentage of Fe, TOF’s are in reasonable agreement with previously reported values where Fe% was measured by ICP-MS (Figure S18).^18^ Yet, it is important that our work has shown dynamic increases in TOF numbers normalized against the amount of detectable active sites, instead of normalizing against a fixed atomic amount.

## Conclusions

In summary, our spectroscopic studies show clear evidence of dynamic accumulation of oxidized Ni centers at OER potential, irrespective of surface Fe incorporation. The rate of discharge of these Ni centers increases at higher oxidative potentials, with the discharge rate observed from open-circuit decay being significantly larger in the presence of surface-incorporated Fe. These results point toward a mechanism that relies on a large fraction of Ni sites getting oxidized to form NiOO at potentials >1.4 V_RHE_ as charge reservoirs, and the OER kinetics being dominated by a smaller fraction of Fe species in the vicinity of Ni, as examined previously, which may form FeO_*x*_ surface clusters on the NiOOH.^18^ Further increase of OER kinetics based on this mechanism thus requires the formation of NiOO species at low overpotential (resulting in an electronically conductive scaffold^44^), sufficient transfer of holes to the Fe site from the charge reservoir NiOO sites, and fast OER kinetics on the Fe site. It is important to note that our results support this plausible mechanism as one possible interpretation that is consistent with the spectroelectrochemistry data rather than the only explanation. Our results thus offer a refined perspective on the activity of minority sites within a catalyst system, indicating that charge can be stored across a wide range of sites that act like a charge reservoir, but catalysis ultimately occurs at specific locations, which closely interact with the host structure. Based on this finding, we propose strategies for optimizing the OER under considerations of: (1) the formation of a reservoir of oxidized states, (2) efficient hole transport to the active center, and (3) the optimized electronic and geometric configuration of the active site to maximize the OER kinetics.

## Methods

### Electrode Preparation

The Ni­(OH)_2_ films were deposited using a two-electrode setup in 0.01 M Ni­(NO_3_)_2_·6H_2_O (Sigma-Aldrich, 99.999% trace metals basis) in 18.2 MΩ·cm DI water onto an FTO glass substrate. A Pt foil, cleaned in aqua regia, was used as the counter electrode, and a PTFE beaker (VWR), cleaned in 10% H_2_SO_4_ then rinsed with DI water, was used for deposition. The FTO glass substrate was cleaned by subsequent sonication in DI water, isopropanol, and then acetone. −0.15 mA cm^–2^ @ 150 s was applied across the two electrodes using an Autolab PGSTAT204 potentiostat.^6^ The Fe-spiked samples were prepared using the protocol by Ou et al.^18^ In brief, the Fe-spiking solution was prepared by titrating 0.1 mM Fe­(NO_3_)_3_·9H_2_O with HNO_3_ to achieve pH ∼2 (to stabilize against precipitation). Then, 175 μL of the spiking solution was added to ∼10 mL of 1.0 M Fe-free KOH while holding the Fe-free NiO_*x*_H_*y*_ sample under chronoamperometry at 1.55 V_RHE_ for 30 min (Figure S5). Mechanical agitation was maintained during spiking to ensure sufficient mass transport.

### Sample Characterizations

The films were characterized using a scanning electron microscope (SEM, Figure S1) and X-ray photoelectron spectroscopy (XPS, Figure S2). Near-edge X-ray absorption fine structure (NEXAFS, Figure S3) in total electron yield (TEY) and total fluorescence yield (TFY) modes was carried out on the B07 beamline at Diamond Light Source. Characterization details can be found in the Supporting Information.

## Supplementary Material



## Notes

Additional characterizations, electrochemistry measurements, spectroelectrochemistry analysis, kinetics, and mechanism analysis (PDF)

## ○

Current Address: Division of Chemistry and Chemical Engineering, California Institute of Technology, Pasadena, California 91125, United States

## Abbreviations

OER: oxygen evolution reaction
XAS: X-ray absorption spectroscopy
TOF: turnover frequency
SEC: spectroelectrochemistry
XPS: X-ray photoelectron spectroscopy
SEM: scanning electron microscopy
Rds: rate-determining step
NEXAFS: near-edge X-ray fine structure
TEY: total electron yield
TFY: total fluorescence yield
EXAFS: extended X-ray absorption fine structure
