# Supplementary material for: Spectroelectrochemical Studies of Oxygen Evolution Reaction Kinetics for Surface-Incorporated Iron in Nickel Oxyhydroxide Electrocatalysts
Source: ACS Catal. 2026 Mar 19;16(7):6749–57. doi: 10.1021/acscatal.5c09080 (PMC13054788; doi:10.1021/acscatal.5c09080)
Supplement: Supplementary file 1 [file cs5c09080_si_001.pdf]

Supporting Information

Spectroelectrochemical Studies of Oxygen Evolution Reaction Kinetics for Surface Incorporated Iron in Nickel Oxyhydroxide Electrocatalysts

Yifeng Wang,[1] Liam Twight,[2] Nicole Sagui,[2] Minkyoung Kwak,[2] Shannon Boettcher,[2,3,4] Benjamin S. Moss,[5,a *] Ifan E. L. Stephens,[1] James R. Durrant,[5,6 *] Reshma R. Rao[1,6,7 *]

[1] Department of Materials, Imperial College London, London SW7 2AZ, U.K
[2] Department of Chemistry and Biochemistry, University of Oregon, Eugene, Oregon 97403, United States
[3] Department of Chemical & Biomolecular Engineering and Department of Chemistry, University of California, Berkeley, California 94720, United States
[4] Energy Storage and Distributed Resources Division, Lawrence Berkeley National Laboratory, Berkeley, California 94720, United States
[5] Department of Chemistry, Imperial College London, London W12 0BZ, U.K
[6] Centre for Processable Electronics, Imperial College London, London W12 0BZ, U.K
[7] Grantham Institute – Climate Change and the Environment, Imperial College London, South Kensington Campus, London SW7 2AZ, U.K.

[a] Current Address: Division of Chemistry and Chemical Engineering, California Institute of Technology, Pasadena, California 91125, United States
[*] Corresponding authors: benmoss@caltech.edu, j.durrant@imperial.ac.uk, reshma.rao@imperial.ac.uk

Sample Preparation

The $Ni(OH)_2$ films were deposited using a two-electrode setup in 0.01 M $Ni(NO_3)_2 \cdot 6H_2O$ (Sigma-Aldrich, 99.999% trace metals basis) in 18.2 MΩ·cm DI water onto a FTO glass substrate. A Pt foil cleaned in aqua regia was used as the counter electrode, and a PTFE beaker (VWR) cleaned in 10% H_2SO_4 then rinsed with DI water was used for deposition. The FTO glass substrate was cleaned by subsequent sonication in DI water, isopropanol then acetone. −0.15 mA cm^{-2} @ 150 s was applied across the two electrodes using a Autolab PGSTAT204 potentiostat. Fe-spiking solution was prepared by titrating 0.1 mM $Fe(NO_3)_3$ (Sigma-Aldrich, 99.999% trace metals basis) with HNO_3 to achieve pH ~2, which was added to the electrolyte (~0.1 ppm) or deposition solution (50%).[1]

Sample Characterisation

Scanning electron microscopy (SEM) measurements were done on Zeiss Sigma 300 (**Figure S1**). The surface structures detected were in agreement with previous studies.[1,2]

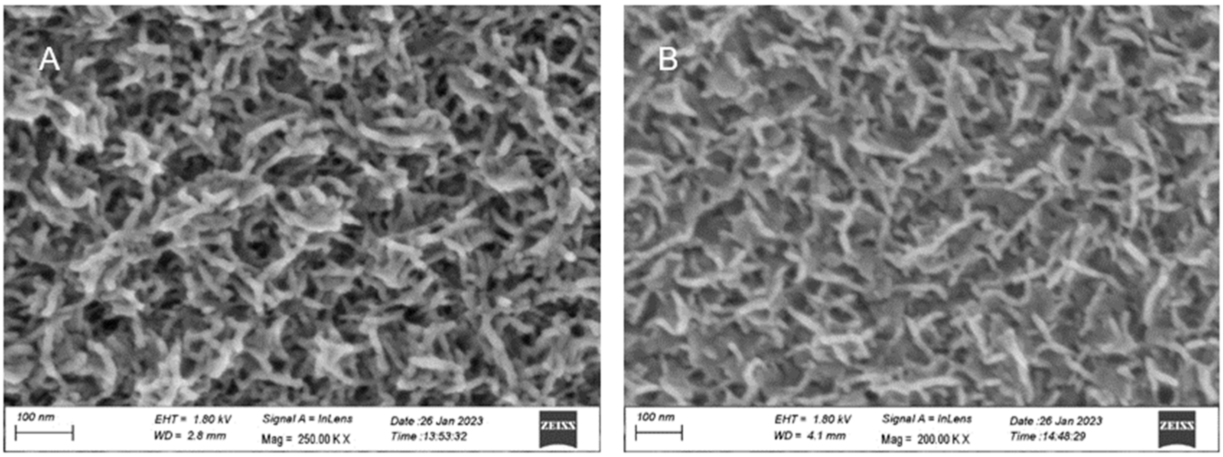

Figure S1: *SEM image of **A**: the as deposited Fe-free NiO_xH_y sample on FTO and **B**: after the Fe spiking and cycled.*

X-ray photoelectron spectroscopy (XPS, **Figure S2**) measurements were carried out with magnesium source. Fe was not detectable in the Fe-free sample as expected. The Fe-spiked samples also do not show an Fe signal, due to the very small amount of Fe incorporated in the surface.

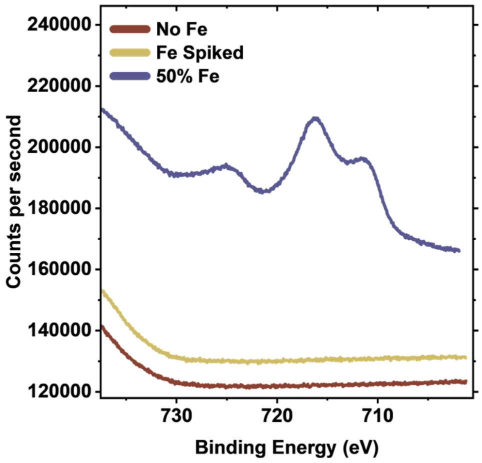

Figure S2: *XPS spectrum of the Fe-free NiO_xH_y, Fe-spiked NiO_xH_y and co-deposited $Ni_{0.5}Fe_{0.5}O_xH_y$ sample on FTO*

Near edge X-ray absorption fine structure were collected in total electron yield (TEY, surface sensitive) and total fluorescence yield (TFY, bulk sensitive). As shown in **Figure S3A**, the TEY mode (probing depth 0 ~ 4 nm [1,2]) reveals no detectable Fe signal in the Fe-free NiO_xH_y. In contrast, the co-deposited $Ni_{0.5}Fe_{0.5}O_xH_y$ exhibits the strongest Fe absorption features, while the Fe-spiked NiO_xH_y shows a weaker but noticeable Fe signal.

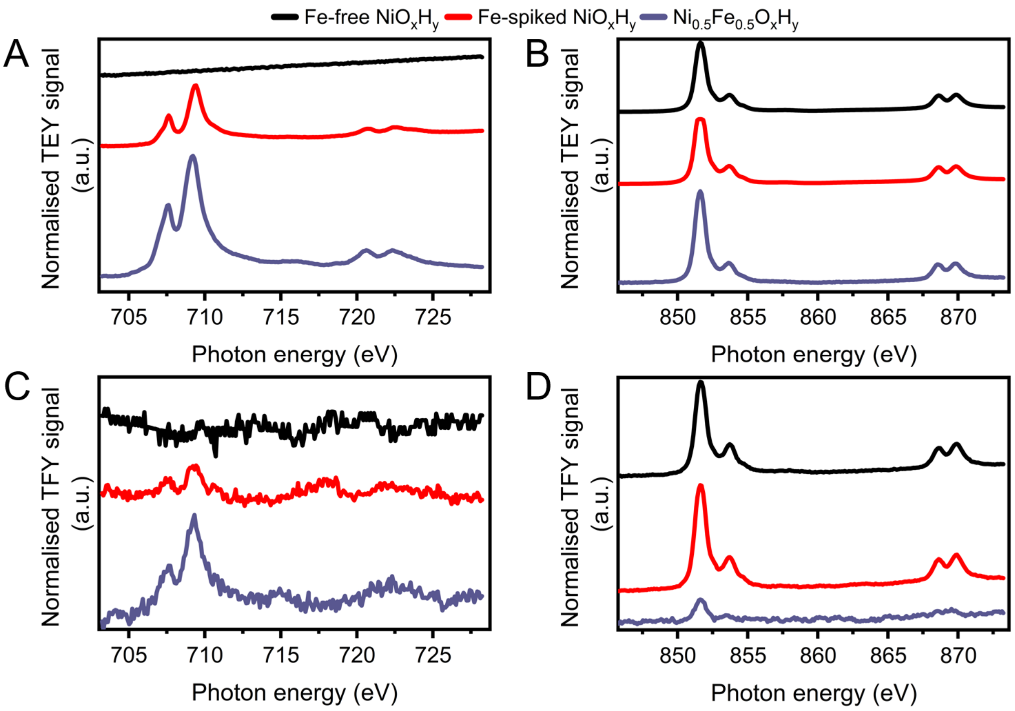

Figure S3: *Near edge X-ray absorption fine structure (NEXAFS) collected in total electron yield (TEY, surface sensitive) and total fluorescence yield (TFY, bulk sensitive) modes for Fe-free NiO_xH_y, Fe-spiked NiO_xH_y and co-deposited $Ni_{0.5}Fe_{0.5}O_xH_y$. Spectra were recorded at Fe L-edge (**A, C**) and Ni L-edge (**B, D**).*

In total fluorescence yield (TFY) mode (**Figure S3C**, probing depth > 100 nm [1,2]), the Fe-free NiOxHy again shows no detectable Fe signal. The Fe-spiked NiO_xH_y displays only a very weak Fe signal, consistent with Fe being primarily confined to the near-surface region. The co-deposited $Ni_{0.5}Fe_{0.5}O_xH_y$ shows the highest Fe intensity in TFY, reflecting its bulk Fe incorporation. Taken together, these results confirm the absence of Fe in the Fe-free NiOxHy and indicate that Fe in the Fe-spiked NiOxHy is predominantly surface-restricted. The Ni L-edge data are shown in **Figure S3B, D**.

The film thickness can be measured by profilometry. As shown in **Figure S4**, the average thicknesses are: NiO_xH_y: ~24.2 nm, $Ni_{0.5}Fe_{0.5}O_xH_y$: ~24.1 nm.

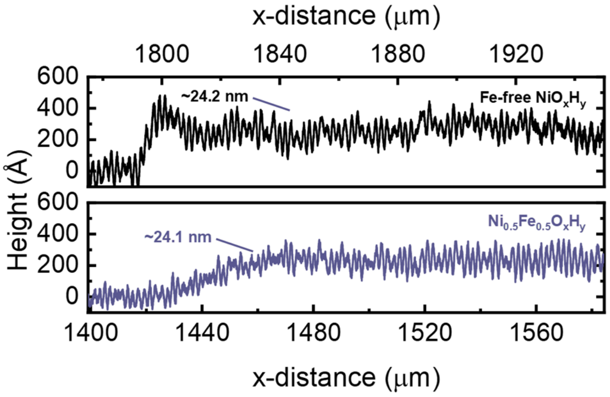

Figure S4: *Profilometry of the Fe-free NiO_xH_y sample and the co-deposited $Ni_{0.5}Fe_{0.5}O_xH_y$*

Electrochemistry Measurements

1.0 M KOH (Sigma-Aldrich, 99.995 Suprapur®) was purified with the method previously described by Trotochaud et al.[1] ~2 g of $Ni(NO_3)_2 \cdot 6H_2O$ (Sigma-Aldrich, ≥99.999% trace metal basis) and ~4 mL of ultra-pure water were added to the tube together with 30 ml of 1 M KOH (≥99.995%, Sigma-Aldrich). The mixture was shaken and sonicated until no visible clumps were present, followed by centrifugation (7000 rpm, 3 min) and subsequent discarding of the supernatant. The green precipitate underwent three washing cycles (~22 mL ultra-pure water and ~ 2 mL unpurified 1 M electrolyte). Afterwards, the tube was filled with ~45 mL of 1 M KOH, shaken until no visible clumps were present, sonicated for 20 minutes and left to rest overnight. The purified 1 M electrolyte was collected by centrifuging (8500 rpm, 25 min), followed by syringe-filtering the supernatant.

A Hg/HgO reference electrode (ALS, RE-2BP) filled with the same 1.0 M purified KOH was used as the reference electrode, and a Pt mesh (ALS) cleaned in the same way descried above was used as the counter electrode. All electrochemical measurements were made in a customised PEEK cell. Potentioelectrochemical impedance spectroscopy (PEIS) was measured at the open circuit potential from, scanned from 0.1 Hz to 100 kHz. Fe-spiking was done by adding 0.1 mM $Fe(NO_3)_3{\cdot}9H_2O$ (pH preadjusted to ~2 with HNO_3) to the Fe-free KOH to yield a relative concentration of Fe at ~ 0.1 ppm, then the Fe-free sample was held at 1.55 V_{RHE} for 30 minutes with mechanical agitation (**Figure S3**), as described by our previous work.[3]

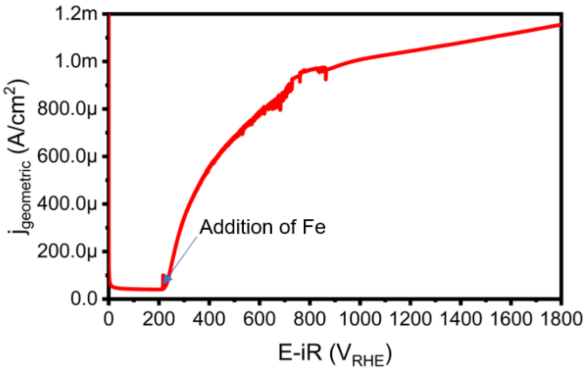

***Figure S5**: Chronoamperometry of the Fe spiking process*

As shown in **Figure S6**, the amount of Ni on the film before and after the spiking process could be estimated via integrating the redox peak. The amount of Ni does not exibit a significant change, although a small mechanical loss might have happned.

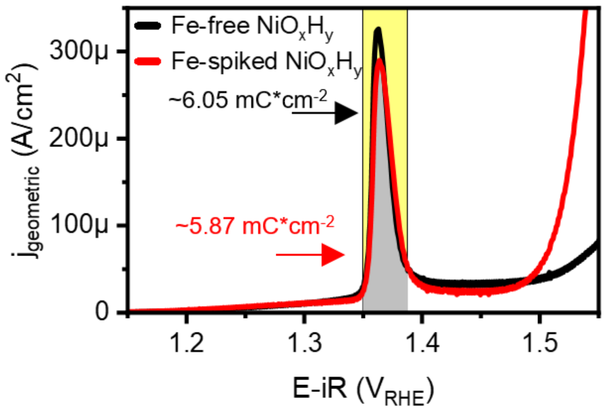

***Figure S6:** Integration of the Ni(OH)2 → NiOOH oxidation peak, 1 mV/s scan rate was assumed*

Spectroelectrochemistry

Operando UV-Vis spectroscopy was carried out in a transmission mode as descried in our previous work. [4,5] Briefly, collimated light from a light source (SLS201L, Thorlabs) was transmitted through a custom-built PEEK cell then collected by a liquid light guide (Edmund optics). Light was then collimated and refocused into a spectroscope ((Kymera 193i, Andor) and a deep-cooled CCD camera (iDus Du420A-BEX2-DD, Andor). Spectra were obtained by scanning continuously under potentiostatic mode at a scan rate of 1 mV/s.

Analysis of the spectra was the same as our work before. [4] Briefly, the difference in absorbance with respect to the resting potential of the sample (1.124 V_{RHE}), $\Delta A(\lambda, U)$. The $\Delta A(\lambda, U)$ spectra can be interpreted as the sum of product of the population of redox species Q_i expressed in density of charge in C/cm^2, and the α_i is the coulombic differential attenuation coefficient calculated from the optical spectroscopy in C^{-1}cm^2 over all the redox transitions i happening over the potential region measured:

$$\Delta A(U, \lambda) = \sum_i Q_i(U) * \Delta\alpha_i(\lambda) \qquad (S1)$$

The difference in absorption spectrum measured at two different potentials $\Delta A(U_1, \lambda) - \Delta A(U_2, \lambda)$ represents a distinct redox transition, and if $\Delta A(U_1, \lambda) - \Delta A(U_2, \lambda)$ normalised by its maximum value is the same across a certain potential region (**Figure S7**) is the same, that means in this potential region only one redox change is happening.

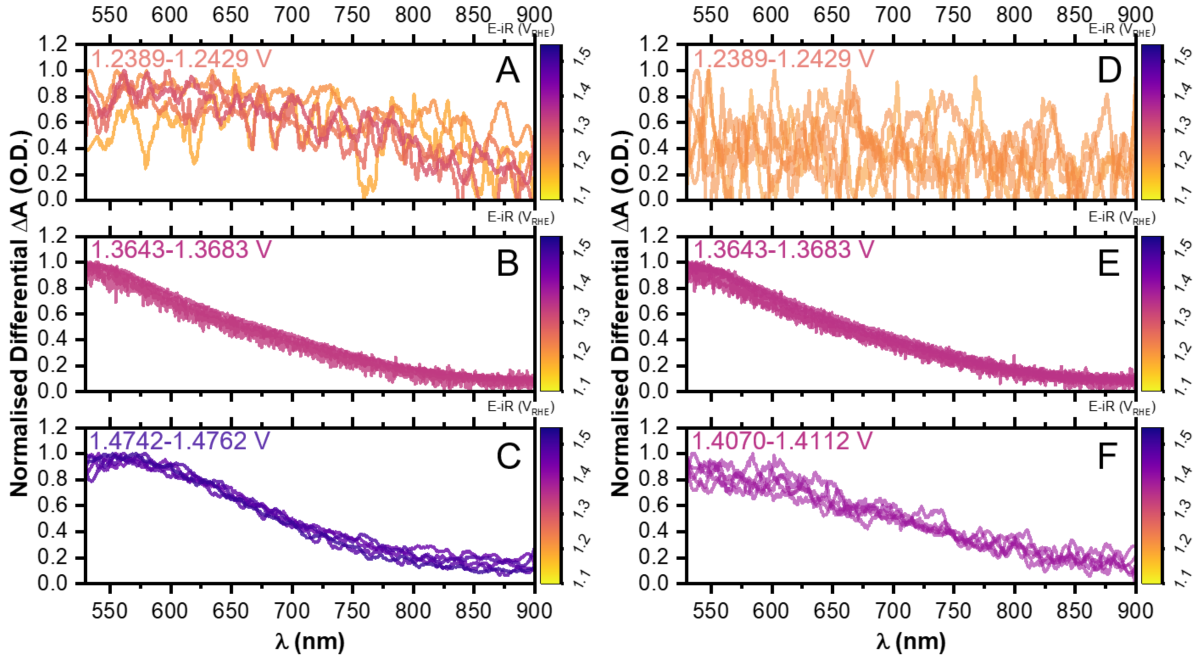

Figure S7: *Normalised differential spectrum of increasing potential windows where only one distinctive absorption signature growth can be detected, plotted for Fe-free NiO_xH_y* ***(A-C)*** *and Fe-spiked NiO_xH_y* ***(D-F)***

As seen in **Figure S7**, there are 3 redox transitions happening across the potential range, as there are 3 distinctive absorption features.

Assuming in a potential region where only the redox transition i where species A transforms into species B happens, the difference in absorption signal between B and A is $\Delta\alpha_i(\lambda)$, which can be normalised by its maximum absorption value at λ_{peak} to yield a normalized differential increment spectrum $\overline{\Delta\alpha_i(\lambda)}$:

$$\overline{\Delta\alpha_i(\lambda)} = \frac{\Delta\alpha_i(\lambda)}{\Delta\alpha_i(\lambda_{peak})} \qquad (S2)$$

$\overline{\Delta\alpha_i(\lambda)}$ is interpreted as the unitless “component spectrum” of a distinct redox transition. Hence summing over all the redox transition measured, the total absorption signal as a function of potential and wavelength can be calculated by:

$$\Delta A(\lambda, U) = \sum_{redox\ transitions\ T_i} Q_i(U) * \Delta\alpha_i(\lambda_{peak}) * \overline{\Delta\alpha_i(\lambda)} \qquad (S3)$$

Since the component spectra of the same redox transition can be taken at a range of potentials hence slightly different due to instrument limits, an average spectrum (**Figure S8**) was calculated using $\overline{\Delta\alpha_i(\lambda)}$ measured at 6 consecutive potentials.

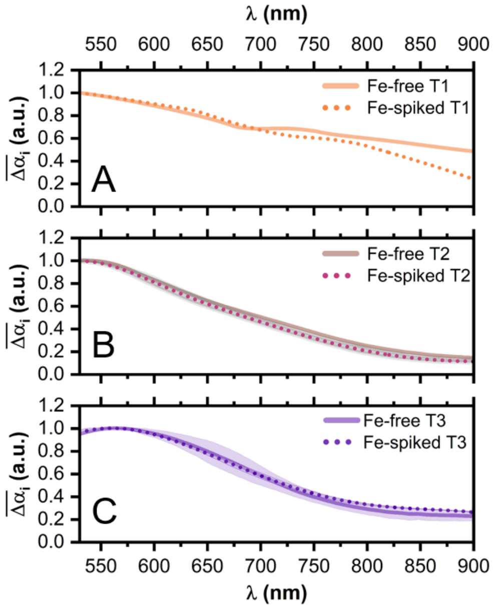

***Figure S8**: Deconvoluted component spectra $\overline{\Delta\alpha_i(\lambda)}$ of T1**(A)**, T2**(B)** and T3**(C)** of of Fe-free and Fe-spiked NiO_xH_y; Mean spectra is plotted for T2 and T3 in lines and dots and the error band is calculated with 95% confidence interval (mean ± 2* standard deviation) across 6 consecutive potentials (1 mV gap). Due to the small amplitude and signal-to-noise ratio of T1, only 1 spectrum was plotted.*

Attenuation Coefficient Measurements

As mentioned above, in the potential regions where only one redox transition contributes to the current such that:

$$\delta\Delta A(\lambda) = \delta Q_i * \Delta\alpha_i(\lambda) \qquad (S4)$$

The equation can be rearranged to:

$$\frac{\delta Q_i}{\delta\Delta A(\lambda)} = \frac{1}{\overline{\Delta\alpha_i(\lambda)} * \Delta\alpha_i(\lambda_{peak})} \qquad (S5)$$

By holding the system at a lower potential first to accumulate the previous state (NiOOH for example for T3), an oxidative potential was then applied to generate oxidized species (NiOO for T3). Specifically, the films were first held at (a) 1.11 V_{RHE} for attenuation coefficient of $Ni(OH)_2$ (b) 1.12 V_{RHE} for attenuation coefficient of NiOOH (c) 1.42 V_{RHE} for attenuation coefficient of Fe-free NiOO and (d) 1.40 V_{RHE} for attenuation coefficient of Fe-spiked NiOO. The oxidising potentials that the samples were held at are denoted on the figure. $\delta\Delta A(\lambda)$ can be measured by the change in optical density at λ_{peak}, and δQ_i can be measured through integrating the current versus time curve of the reduction wave when the system is switched to the lower potential.

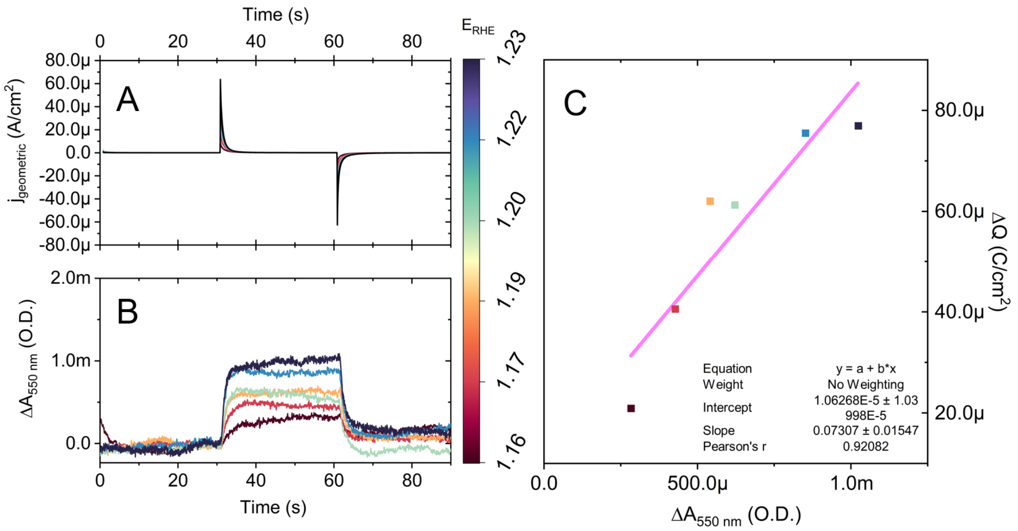

Figure S9: *Attenuation coefficient measurements for Ni(OH)*$_2$*;* ***A:*** *Measured current density vs time;* ***B:*** *Measured optical density at 550 nm (T1) vs time;* ***C:*** *Change in charge during the reduction wave vs change in optical density at 550 nm (T1)*

Due to the insignificance of T1 towards the optical density, and more importantly to avoid perturbing the system too much after the Fe spiking, which might impact the surface structure, the Attenuation coefficients of T1 of Fe-free NiOOH and Fe-spiked NiOOH are assumed to be the same.

There is an irreversibility of optical signal observed for T2 during the measurements (**Figure S10**), which we attributed to the irreversible oxidation of Ni(OH)$_2$ to NiOOH, which is likely due to the poor conductivity of the substrate and the sample. Hence attenuation coefficient of T2 is estimated with the charging current so that it matches the magnitude measured in linear sweep voltammogram.

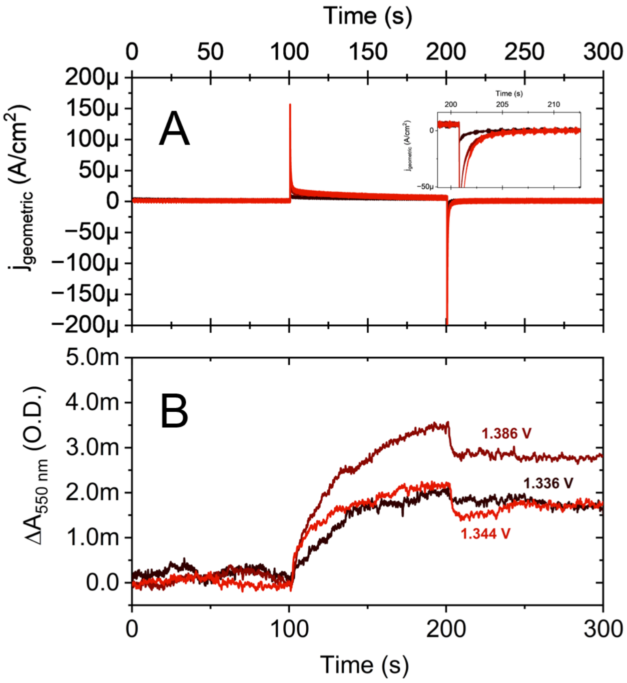

Figure S10: *Attenuation coefficient measurements for NiOOH;* ***A:*** *Measured current density vs time;* ***B:*** *Measured optical density at 550 nm (T2)*

To verify the validity of this value, we can compare the charge passed in the redox wave for the measured cyclic voltammogram and from the cyclic voltammogram obtained based on the fitting of the optical signal. As demonstrated in the charge determined during the NiOOH + OH^- → NiOO + H_2O + e^- process from the fitting of the optical spectroscopy data (~5 mC*cm^{-2}, **Figure S13B**) agrees well with the charged obtained from redox wave integration from the linear sweep voltammogram (~6 mC*cm^{-2}, **Figure S6**).

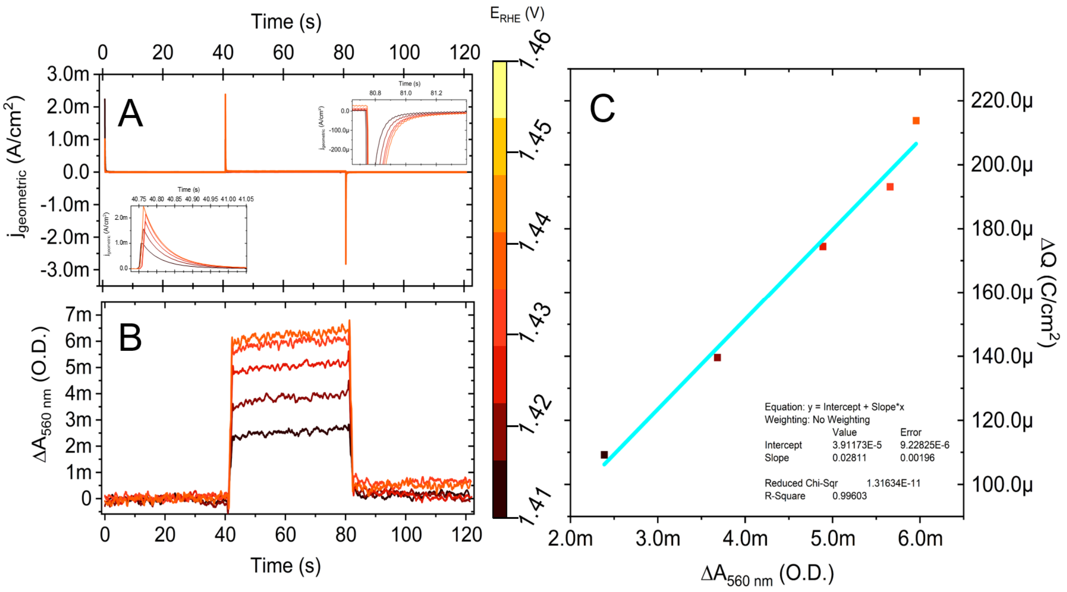

Figure S11: *Attenuation coefficient measurements for Fe-free NiOO;* ***A:*** *Measured current density vs time;* ***B:*** *Measured optical density at 560 nm (T3) vs time;* ***C:*** *Change in charge during the reduction wave vs change in optical density at 560 nm (T3)*

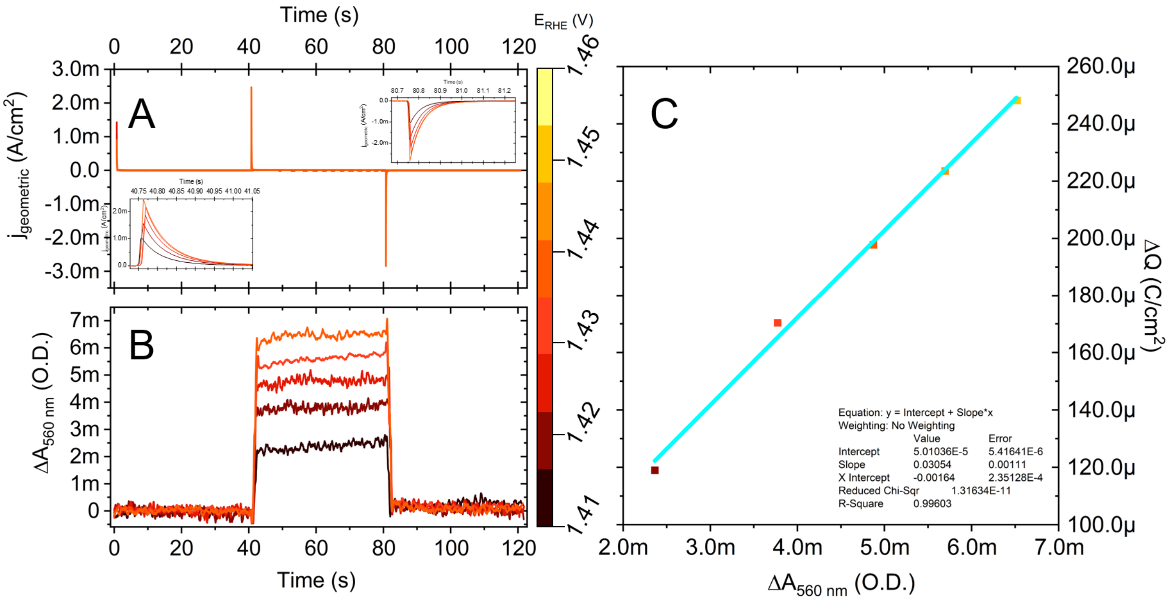

Figure S12*: Attenuation coefficient measurements for Fe-spiked NiOO;* ***A:*** *Measured current density vs time;* ***B:*** *Measured optical density at 560 nm (T3) vs time;* ***C:*** *Change in charge during the reduction wave vs change in optical density at 560 nm (T3)*

The attenuation coefficient measured for T3 are in reasonable agreement with the literature values (~0.035 C*OD^{-1}*cm^{-2} as measured previously on solution combustion synthesised NiO [5] and ~0.038 C*OD^{-1}*cm^{-2} on $Ni_{0.9}Fe_{0.1}O$ nanoparticles [6]).

Spectra Fitting and Deconvolution

Fitting residual is calculated from by subtracting the fitted spectrum by the original spectrum at each potential and wavelength, as described in our previous work.[4,5] Fitted change in optical signal (**Figure S13A**) is converted to calculated change in population of species accumulated (**Figure S13B)** using the attenuation coefficients calculated from above.

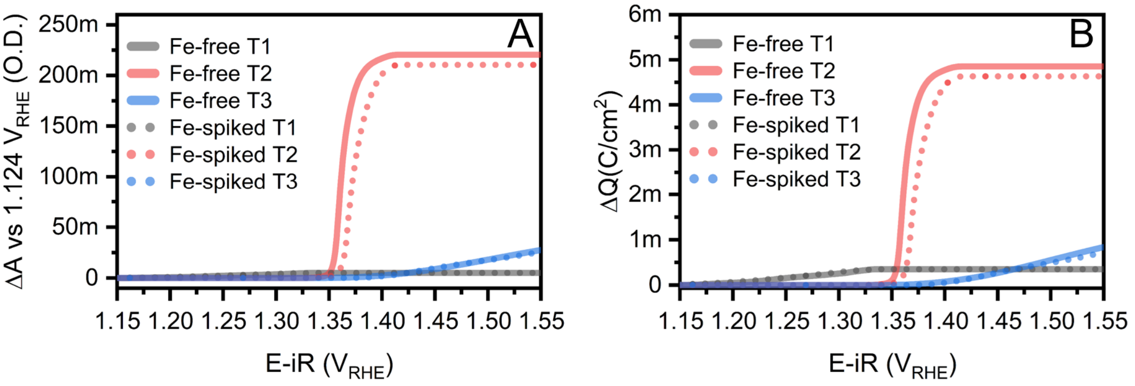

***Figure S13**: Fitted change in optical signal **(A)** and calculated change in population of species accumulated **(B)** during T1-T3 of the Fe free and Fe spiked NiO_xH_y*

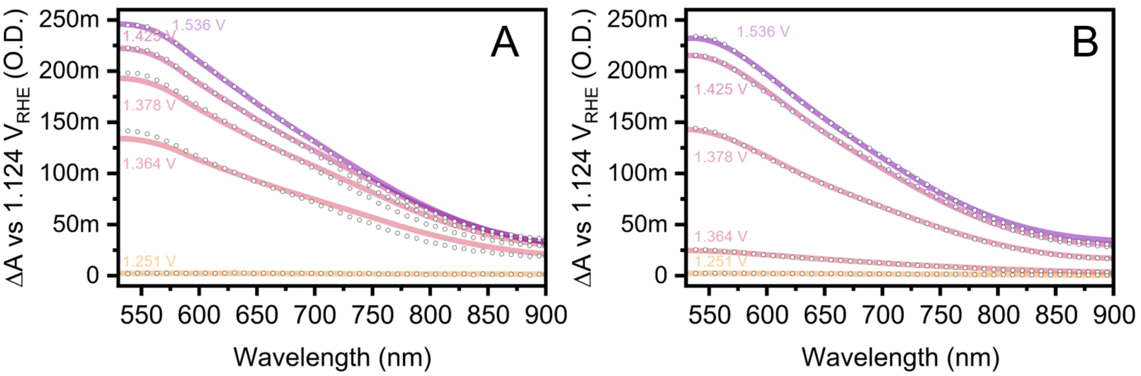

***Figure S14**: Spectra reconstructed from the fitting (dotted) versus the original spectra (solid) of change in optical signal as a function of the Fe-free **(A)** and Fe spiked **(B)** NiO_xH_y*, plotted at several potentials

The good agreement between the reconstructed spectra and the original spectra supports the correctness of the model (**Figure S14**). The residual of fitting is calculated by subtracting the original spectrum from the fitted spectrum (**Figure S15**). The residual spectra are small in magnitude which suggests good fitting.

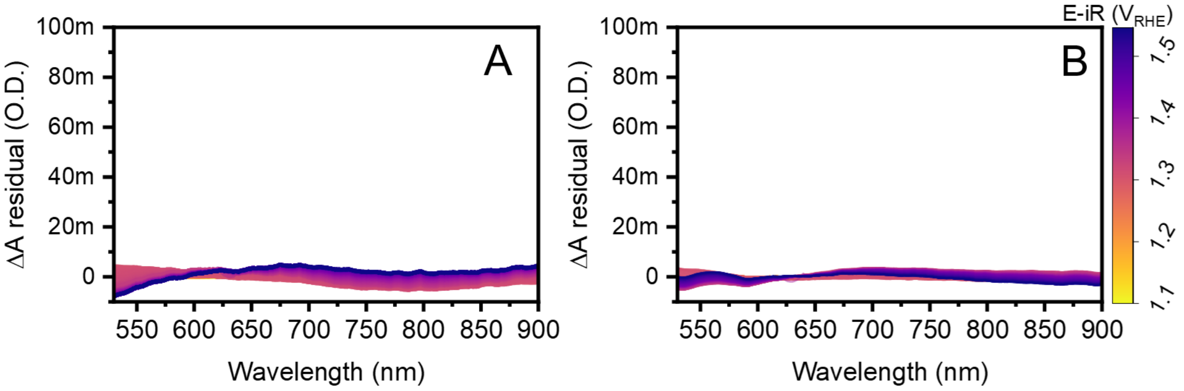

***Figure S15**: Fitting residuals of the Fe-free **(A)** and Fe spiked **(B)** NiO_xH_y*

The fitting residues are insignificant compared to the detected signal (8 mO.D. (maximum residue absorbance) / 250 mO.D. (maximum real spectrum absorbance) =3% for the Fe-free NiO_xH_y and 5 mO.D. / 240 mO.D.= 2% for the Fe-spiked NiO_xH_y), hence we believe that the error obtained are negligible. Nevertheless, the fitting model agrees well with the potential windows and the species probed by XAS and Raman reported previously [7,8,8,9], as the fitting method has been also demonstrated on other metal oxides.[5,10,11]

To confirm whether Fe oxidation in this case is beyond the detection limit of the instrument, a simple estimation can be conducted.

The sample was deposited with -0.15 mA cm^{-2} for 150 s, which passes a total charge of 0.0225 C cm^{-2}. Assuming a 30% faradic efficiency in the deposition process, then 6.6 mC cm^{-2} of Ni has been deposited on the substrate. Thus, in the $Ni_{0.5}Fe_{0.5}O_xH_y$, 3.3 mC cm^{-2} of Fe was deposited assuming that Ni and Fe deposit at the same rate. The maximum Fe-centred absorption signal at ~600 nm produced by the $Ni_{0.5}Fe_{0.5}O_xH_y$ sample is 30 mOD at 1.55 V_{RHE}, which is 0.11 C $cm^{-2}OD^{-1}$.

The resolution of the instrument is 1 mOD. 1 mOD corresponds to 0.11 mC*cm^{-2} of Fe. This corresponds to $0.11*10^{-3}$ / $726*10^{-6}$ = 15 % of the total catalytically Fe-spiked NiOO at 1.55 V_{RHE}. This value is significantly larger than what we expected for surface restricted Fe. Therefore, we believe that the surface redox of Fe cannot be detected by our instrument.

Additional TOF calculations

Assuming all the nickel sites contribute towards the transition of $Ni(OH)_2 + OH^- \rightarrow NiOOH + H_2O + e^-$, and parts of the NiOOH sites converts into NiOO as a function of potential, the coverage of NiOO over NiOOH (**Figure S16**) can be calculated as:

$$\theta_{NiOO} = \frac{\Delta Q_{NiOO}}{\Delta Q_{NiOOH\,max}} \qquad (S6)$$

Where $\Delta Q_{NiOOH\,max}$ is the maximum change in the population of NiOOH observed.

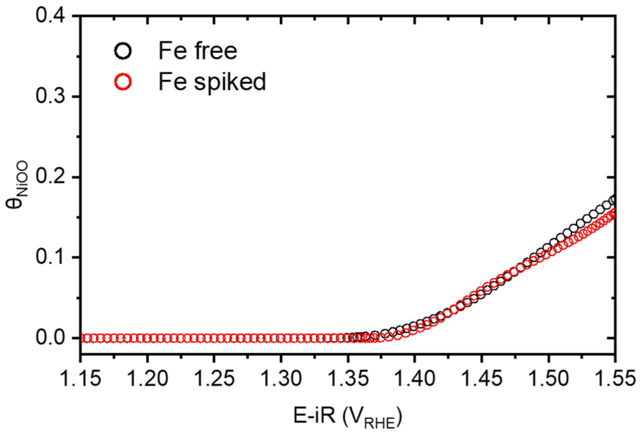

Figure S16: *Coverage of NiOO versus potential applied*

The same TOF_{Ni} values calculated previously in the main text can now be plotted against coverage **(Figure S17)**:

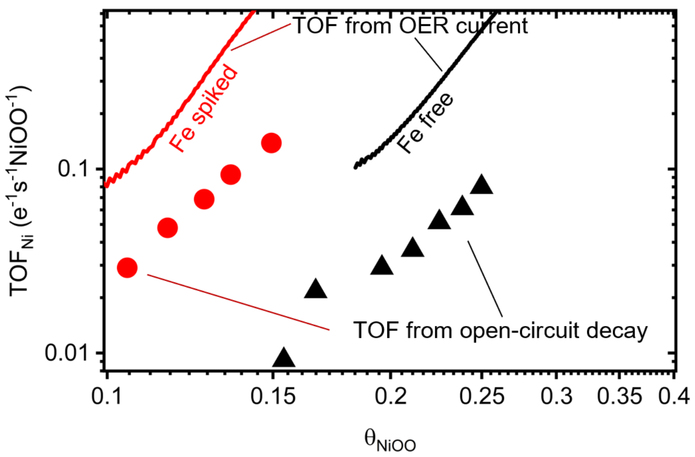

Figure S17: *TOF$_{Ni}$ as a function of the coverage of NiOO*

In addition to the TOF calculations mentioned in the main text, TOF$_{Fe}$ with Fe% determined from inductively coupled plasma mass spectrometry (ICP-MS) from previous work[3] is also plotted for comparison (**Figure S18**). Qualitatively, the orders of magnitudes agree between studies.

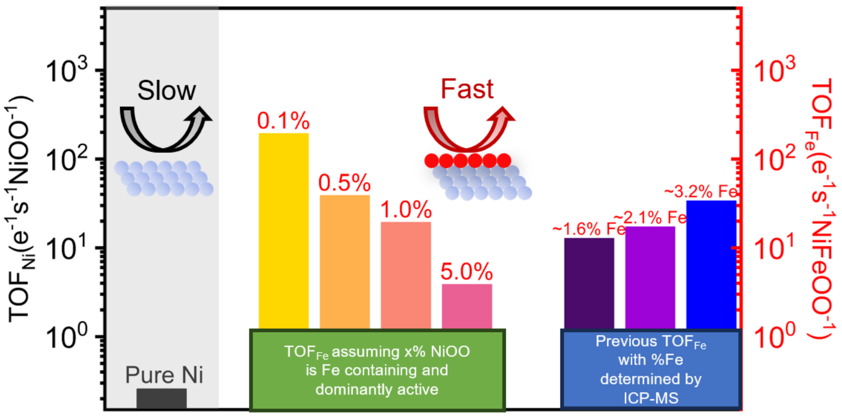

Figure S18: *TOF$_{Ni}$ and TOF$_{Fe}$ at 1.52 V$_{RHE}$ calculated from the Fe spiked NiO$_x$H$_y$, and TOF$_{Fe}$ previously reported by Ou et al. with the Fe% determined by ICP-MS [3]*

References

(1) Trotochaud, L.; Young, S. L.; Ranney, J. K.; Boettcher, S. W. Nickel–Iron Oxyhydroxide Oxygen-Evolution Electrocatalysts: The Role of Intentional and Incidental Iron Incorporation. *J. Am. Chem. Soc.* **2014**, *136* (18), 6744–6753. https://doi.org/10.1021/ja502379c.

(2) *Spectroelectrochemical study of water oxidation on nickel and iron oxyhydroxide electrocatalysts | Nature Communications*. https://www.nature.com/articles/s41467-019-13061-0 (accessed 2024-06-10).

(3) Ou, Y.; Twight, L. P.; Samanta, B.; Liu, L.; Biswas, S.; Fehrs, J. L.; Sagui, N. A.; Villalobos, J.; Morales-Santelices, J.; Antipin, D.; Risch, M.; Toroker, M. C.; Boettcher, S. W. Cooperative Fe Sites on Transition Metal (Oxy)Hydroxides Drive High Oxygen Evolution Activity in Base. *Nat Commun* **2023**, *14* (1), 7688. https://doi.org/10.1038/s41467-023-43305-z.

(4) *Cooperative Effects Drive Water Oxidation Catalysis in Cobalt Electrocatalysts through the Destabilization of Intermediates | Journal of the American Chemical Society*. https://pubs.acs.org/doi/full/10.1021/jacs.3c11651 (accessed 2024-07-05).

(5) Rao, R. R.; Corby, S.; Bucci, A.; García-Tecedor, M.; Mesa, C. A.; Rossmeisl, J.; Giménez, S.; Lloret-Fillol, J.; Stephens, I. E. L.; Durrant, J. R. Spectroelectrochemical Analysis of the Water Oxidation Mechanism on Doped Nickel Oxides. *J. Am. Chem. Soc.* **2022**, *144* (17), 7622–7633. https://doi.org/10.1021/jacs.1c08152.

(6) Rao, R. R.; Bucci, A.; Corby, S.; Moss, B.; Liang, C.; Gopakumar, A.; Stephens, I. E. L.; Lloret-Fillol, J.; Durrant, J. R. Unraveling the Role of Particle Size and Nanostructuring on the Oxygen Evolution Activity of Fe-Doped NiO. *ACS Catal.* **2024**, *14* (15), 11389–11399. https://doi.org/10.1021/acscatal.4c02329.

(7) Görlin, M.; Ferreira de Araújo, J.; Schmies, H.; Bernsmeier, D.; Dresp, S.; Gliech, M.; Jusys, Z.; Chernev, P.; Kraehnert, R.; Dau, H.; Strasser, P. Tracking Catalyst Redox States and Reaction Dynamics in Ni–Fe Oxyhydroxide Oxygen Evolution Reaction Electrocatalysts: The Role of Catalyst Support and Electrolyte pH. *J. Am. Chem. Soc.* **2017**, *139* (5), 2070–2082. https://doi.org/10.1021/jacs.6b12250.

(8) Görlin, M.; Chernev, P.; Paciok, P.; Tai, C.-W.; Araújo, J. F. de; Reier, T.; Heggen, M.; Dunin-Borkowski, R.; Strasser, P.; Dau, H. Formation of Unexpectedly Active Ni–Fe Oxygen Evolution Electrocatalysts by Physically Mixing Ni and Fe Oxyhydroxides. *Chem. Commun.* **2019**, *55* (6), 818–821. https://doi.org/10.1039/C8CC06410E.

(9) Görlin, M.; Halldin Stenlid, J.; Koroidov, S.; Wang, H.-Y.; Börner, M.; Shipilin, M.; Kalinko, A.; Murzin, V.; Safonova, O. V.; Nachtegaal, M.; Uheida, A.; Dutta, J.; Bauer, M.; Nilsson, A.; Diaz-Morales, O. Key Activity Descriptors of Nickel-Iron Oxygen Evolution Electrocatalysts in the Presence of Alkali Metal Cations. *Nat Commun* **2020**, *11* (1), 6181. https://doi.org/10.1038/s41467-020-19729-2.

(10) Liang, C.; Rao, R. R.; Svane, K. L.; Hadden, J. H. L.; Moss, B.; Scott, S. B.; Sachs, M.; Murawski, J.; Frandsen, A. M.; Riley, D. J.; Ryan, M. P.; Rossmeisl, J.; Durrant, J. R.; Stephens, I. E. L. Unravelling the Effects of Active Site Density and Energetics on the Water Oxidation Activity of Iridium Oxides. *Nat Catal* **2024**, 1–13. https://doi.org/10.1038/s41929-024-01168-7.

(11) Moss, B.; Svane, K. L.; Nieto-Castro, D.; Rao, R. R.; Scott, S. B.; Tseng, C.; Sachs, M.; Pennathur, A.; Liang, C.; Oldham, L. I.; Mazzolini, E.; Jurado, L.; Sankar, G.; Parry, S.; Celorrio, V.; Dawlaty, J. M.; Rossmeisl, J.; Galán-Mascarós, J. R.; Stephens, I. E. L.; Durrant, J. R. Cooperative Effects Drive Water Oxidation Catalysis in Cobalt Electrocatalysts through the Destabilization of Intermediates. *J. Am. Chem. Soc.* **2024**, *146* (13), 8915–8927. https://doi.org/10.1021/jacs.3c11651.
